# Abscess of Antrum

**Published:** 1883-03

**Authors:** 


### ABSCESS OF ANTRUM.

Dr. C. E. Nelson, of New York, writes : " In bringing the follow-lowing case before the readers of the *Record*, I wish to draw attention to two points: First, the grave mistake in the original diagnosis; and, second, the ingenious nature of the operation which was subsequently performed.

" A gentleman in the prime of life suffered from an extensive swelling of the face, with extrusion of the eyeball, caused by an abscess of the antrum. Believing that the trouble was mainly in the eye, he consulted a celebrated New York oculist, who advised immediate removal of the eyeball. Subsequently he consulted Dr. George P. Miles, a New York dental surgeon, who diagnosed abscess of the antrum from a diseased molar tooth. He believed the eye to be uninjured, and gave it as his opinion that, when the pus was evacuated and the swelling reduced the eye would return to its normal situation.

" Instead of extracting the decayed tooth and puncturing the antrum with a trochar, as is usually recommended in such cases, Dr. Miles drilled through the tooth and socket into the antrum, and through this small opening perfectly evacuated the abscess cavity; the swelling rapidly subsided, and the eyeball returned to its normal position. The decayed tooth was subsequently treated, and the patient eventually made an excellent recovery without losing either eye or tooth. The importance of accurate diagnosis in such a case is self-evident."—*Canada Medical Record, June.*

THE supply of hard and decorative timber for mechanical and artistic purposes will, in the near future, it is thought, be largely obtained from Brazil, where the sources of these choice and valuable materials are known to be well nigh inexhaustible. It is stated that within an area of half a square mile Agassiz counted 117 different kinds of wood, many of them admirably fitted by their hardness, tints and beautiful grains for the finest cabinet work. One of these, familiarly known as tortoise-shell wood, and believed to be the most precious wood in the world, is found in large quantities along the tributaries of the upper Amazon, where the water can be easily used as a motive power.

